# Proteus Syndrome: A Rare Congenital Disorder

**DOI:** 10.7759/cureus.60072

**Published:** 2024-05-10

**Authors:** Sanjay M Khaladkar, Neeha A Jhala, Karishma S Krishnani, Eshan C Durgi

**Affiliations:** 1 Radiodiagnosis, Dr. D. Y. Patil Medical College, Hospital & Research Centre, Dr. D. Y. Patil Vidyapeeth, Pune, IND; 2 Radiodiagnosis, Dr. D. Y. Patil Medical College, Hospital & Research Centre, Dr. D.Y. Patil Vidyapeeth, Pune, IND

**Keywords:** pediatric disease, connective tissue naevus, pediatric vascular malformation, congenital abnormalities, proteus syndrome

## Abstract

An uncommon congenital hamartomatous disorder called Proteus syndrome is characterized by multifocal tissue expansion originating from all three germinal layers. Diagnosis mainly relies on clinical and radiological criteria. Here, we present a case of a 13-year-old female child exhibiting bony, soft tissue, and vascular abnormalities, along with developmental delay. We conclude by highlighting the importance of imaging studies in conjunction with physical examination, which are characterized by general and specific criteria to diagnose this rare condition until a specific gene test becomes available.

## Introduction

Proteus syndrome presents a complex diagnostic challenge due to its rarity and diverse clinical manifestations. This congenital hamartomatous condition leads to multifocal tissue overgrowth from various germinal layers [1]. Initially described by Wiedermann et al. in 1983, further elucidation by Cohen and Hayden has shed light on its clinical spectrum [2, 3]. Although the syndrome is linked to a somatic activating mutation in the AKT1 gene, blood DNA testing is still unable to identify it; thus, clinical and radiological assessments are the only methods available [4]. Manifestations encompass soft tissue, skeletal, vascular, and visceral abnormalities, underscoring the syndrome's systemic impact [5].

In our presented case, soft tissue and skeletal manifestations, along with vascular malformations, were evident, aligning with the syndrome's diverse phenotypic expression. Differential diagnosis emphasizes the significance of precise diagnostic criteria by differentiating it from Klippel-Trenaunay-Weber syndrome and neurofibromatosis type I [6]. Developed by the National Institutes of Health (NIH) in 1998, these criteria, coupled with recommended diagnostic tests such as magnetic resonance (MR) of the abdomen and pelvis, MRI of the brain, and high-resolution computed tomography (HRCT) of the chest, facilitate timely diagnosis [7]. Treatment necessitates a multidisciplinary approach, encompassing orthopedic interventions, growth regulation techniques, and surgical interventions, while vigilant tumor screening remains paramount. By elucidating the complexities of Proteus syndrome and outlining an integrated diagnostic and therapeutic approach, this case report aims to enhance clinical management and patient outcomes [8].

## Case presentation

**Figure 1:**
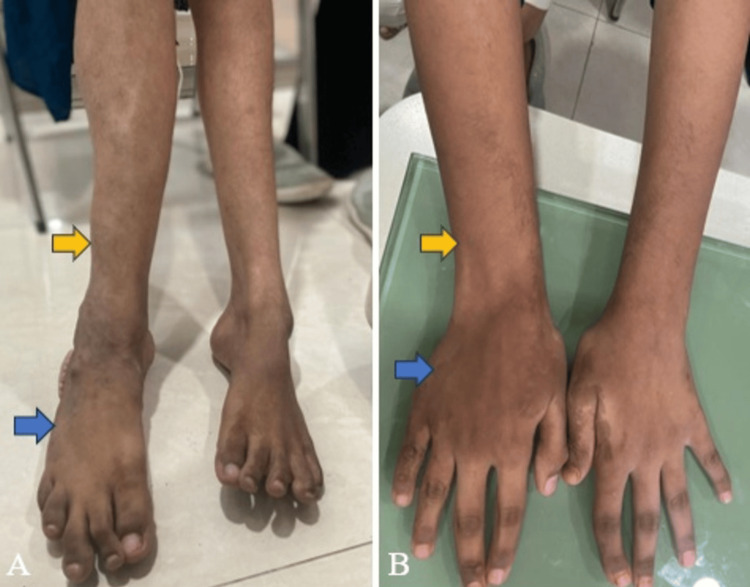
Clinical images show limb size and limb length discrepancies: Image A displays the right leg (yellow arrow) and the right foot (blue arrow), appearing larger than the left; Image B exhibits the right forearm (yellow arrow) and the right hand (blue arrow), appearing larger than the left.

**Figure 2:**
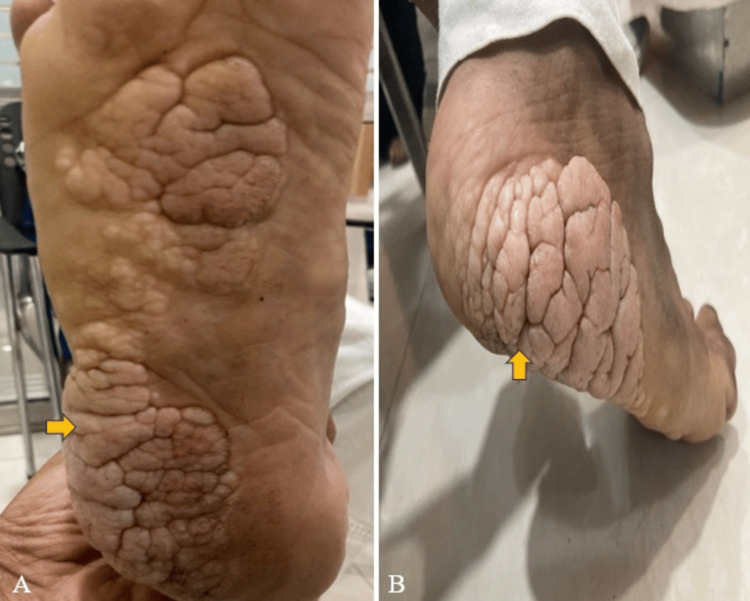
Clinical images depict a cerebriform connective tissue overgrowth in the plantar (A) and lateral (B) aspects of the right foot (yellow arrow).

A 13-year-old female presented with a history of irregular growth on the lateral aspect of the right foot, which began at the age of four and has progressively worsened since. The patient also had a motor developmental delay. Upon clinical examination, inversion of the right foot was observed, along with a limb length discrepancy where the right forearm and hand were longer than the left, and the right foot was longer than the left (Figure 1). A cerebriform connective tissue overgrowth was noted on the plantar and lateral aspects of the right foot (Figure 2). Additionally, there was a history of intrauterine hydrocephalus. The patient was born at term via normal vaginal delivery with a birth weight of 3 kg. No other significant maternal history was noted.

**Figure 3:**
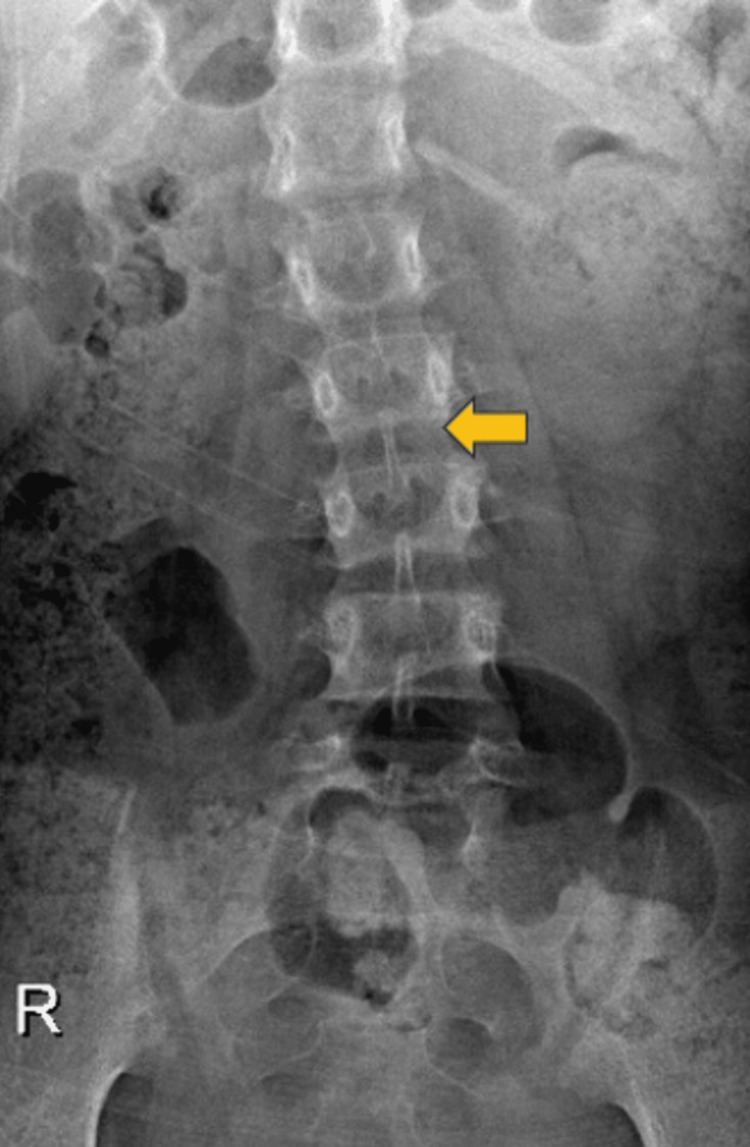
A radiograph (anteroposterior view) of the lumbar spine shows levoconvex scoliosis (yellow arrow).

A radiograph of the lumbar spine revealed levoconvex scoliosis (Figure 3).

**Figure 4:**
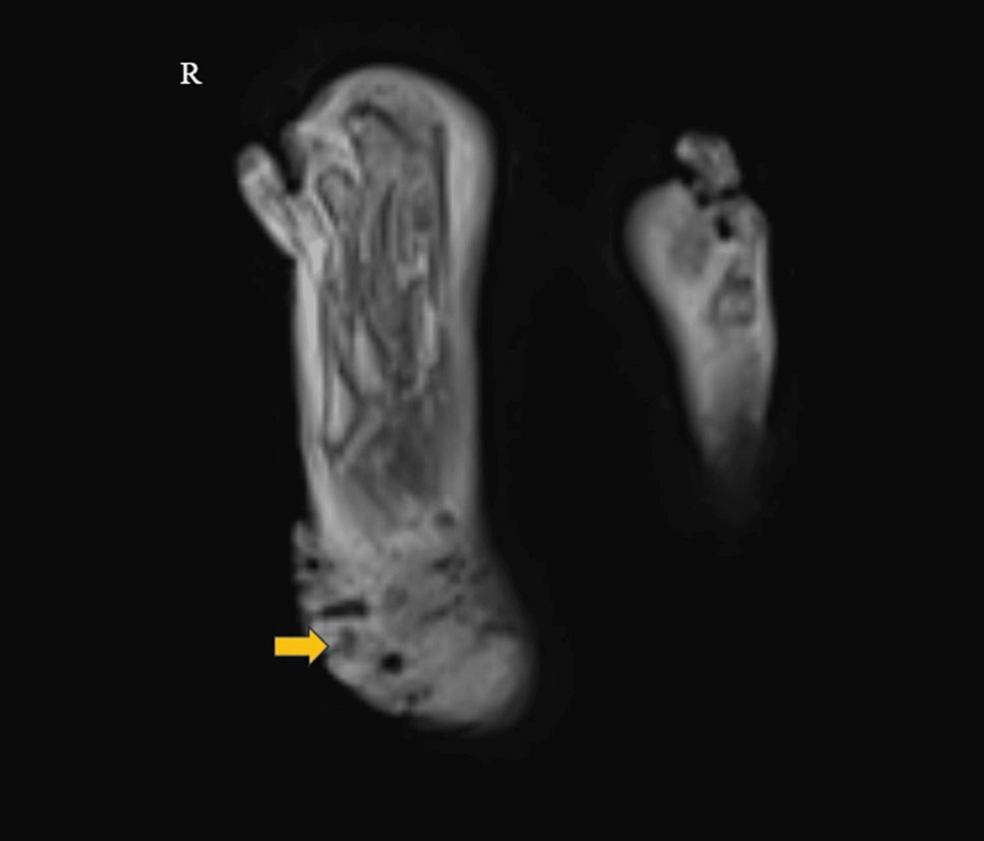
An MRI of the right lower limb axial T1WI shows cerebriform connective tissue overgrowth on the plantar and lateral aspects of the right foot (yellow arrow). T1WI: T1-weighted imaging

**Figure 5:**
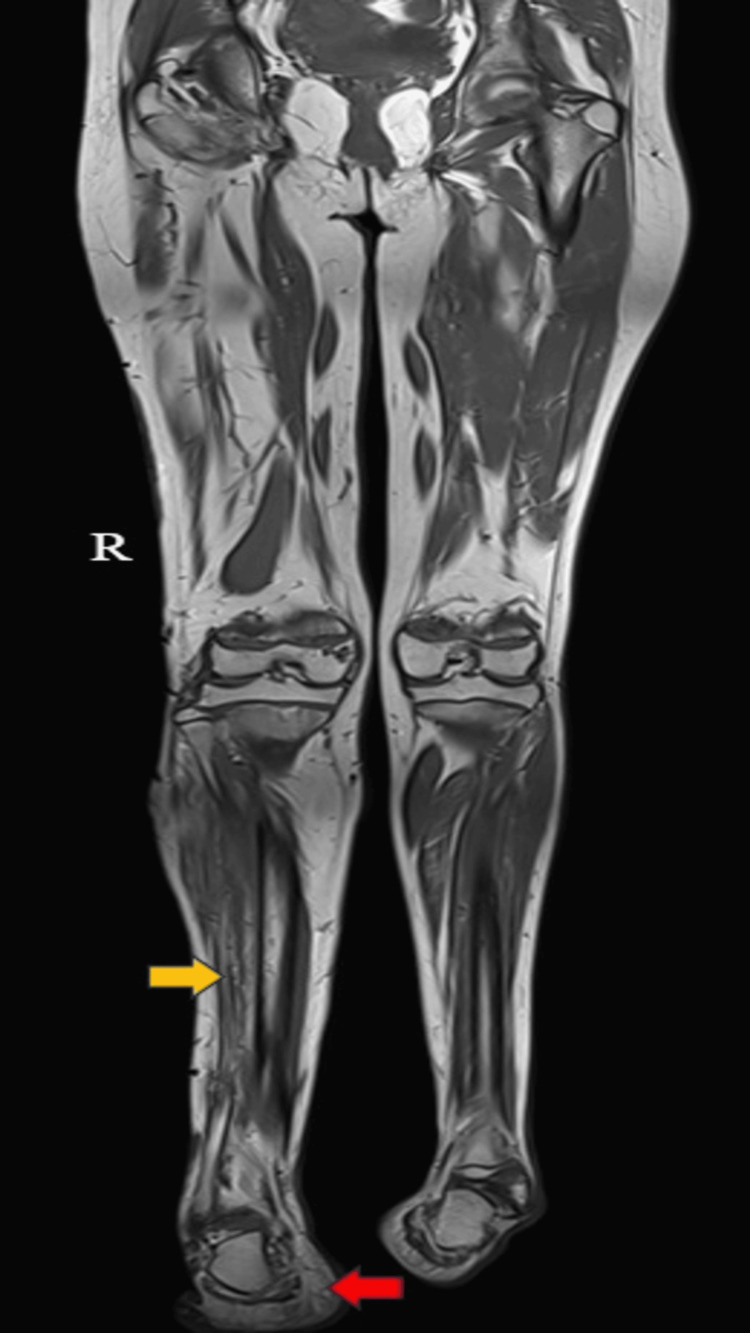
An MRI of the bilateral lower limb axial T1WI shows an enlarged and elongated right lower limb (yellow arrow) and inversion of bilateral feet (red arrow). T1WI: T1-weighted imaging

**Figure 6:**
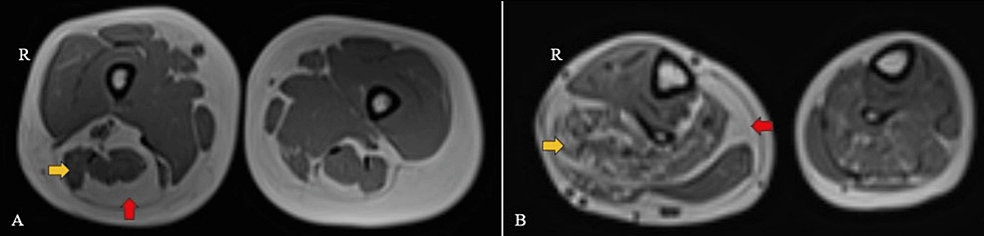
An MRI of the bilateral lower limb (axial, T1WI images) shows muscle atrophy (yellow arrows) accompanied by hypertrophy of the intermuscular and subcutaneous fat planes (red arrows) in the right thigh (A) and leg (B). T1WI: T1-weighted imaging

The MRI findings of the right lower limb revealed several notable abnormalities, which were as follows: On T1-weighted imaging (T1WI), a cerebriform connective tissue overgrowth or nevus was identified on both the plantar and lateral aspects of the right foot, indicating an irregular growth pattern in this region (Figure 4). Additionally, the right foot appeared enlarged in size, suggesting macrodactyly or foot overgrowth. Furthermore, a visible discrepancy in length and size was noted between the affected leg (right) and the contralateral limb (left), indicating asymmetrical growth (Figure 5). In the thigh, muscle atrophy was evident, accompanied by hypertrophy of the intermuscular and subcutaneous fat planes, which may have contributed to the observed differences in leg size. Similarly, hypertrophy of subcutaneous fat and fatty infiltration within the calf muscles were observed (Figure 6). Moreover, both fibulas displayed inward bowing, indicating structural abnormalities in the lower limb bones. Overall, these MRI findings underscored the complex nature of the patient's condition, which was characterized by asymmetrical growth patterns and structural anomalies throughout the right lower limb.

**Figure 7:**
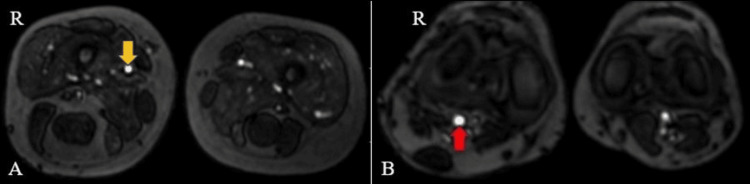
Contrast-enhanced magnetic resonance angiogram images of the bilateral thigh (A) and leg (B) show enlarged right superficial femoral (yellow arrow) and popliteal arteries (red arrow).

**Figure 8:**
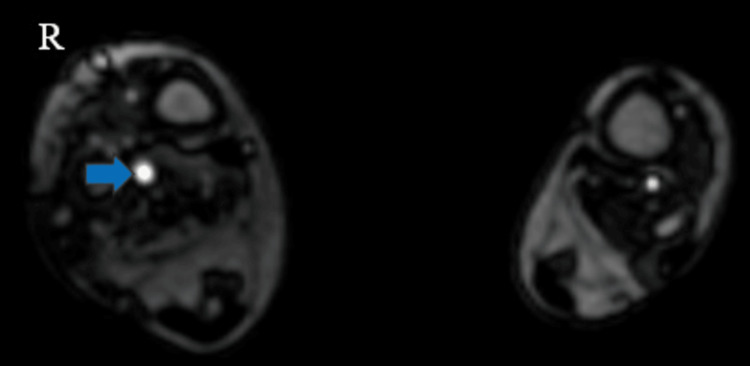
Contrast-enhanced magnetic resonance angiogram images of the bilateral lower limb show an enlarged right posterior tibial artery (blue arrow).

**Figure 9:**
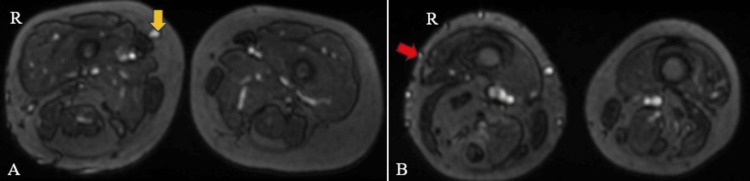
Contrast-enhanced magnetic resonance venogram images of the bilateral lower limb show an enlarged right long saphenous vein (yellow arrow) (A) and varicosities (red arrow) in the anterior and lateral aspects of the right thigh region (B).

**Figure 10:**
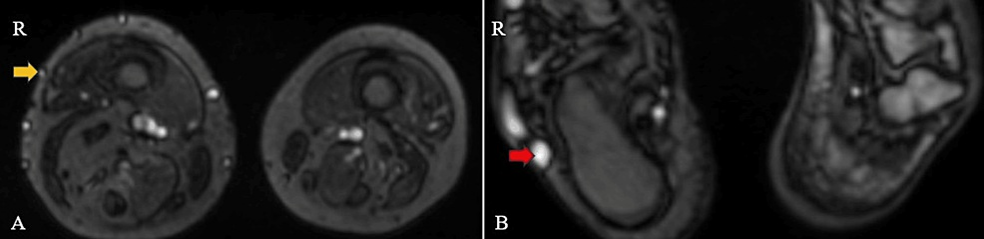
Contrast-enhanced magnetic resonance venogram images of the bilateral lower limb show varicosities in the anterior and lateral aspects of the right leg (yellow arrow) (A) and the lateral aspect of the right foot (red arrow) (B).

On right lower limb contrast-enhanced angiography and venography, several significant findings were observed. Enlargement was noted in the right superficial femoral, popliteal, and posterior tibial arteries, indicating increased arterial size in these regions (Figures 7, 8). Additionally, feeders originating from the posterior tibial artery were identified in the lower third of the leg, alongside prominent vascular channels on the posterolateral aspect of the right leg and foot, as well as abnormal vessels within the foot itself. Furthermore, dilation was observed in the lower third of the superficial femoral and popliteal veins. A prominent long saphenous vein was also evident (Figure 9). Multiple varicosities were detected along the anterior and lateral aspects of the thigh (Figure 9), as well as the anterior, lateral, and posterior aspects of the leg within the subcutaneous plane (Figure 10). These findings collectively suggest a complex vascular condition characterized by arterial enlargement, vascular malformations, venous dilation, and varicosities throughout the right lower limb.

The diagnosis of Proteus syndrome was made based on the clinical examination and radiological evaluation, in accordance with both general and specific criteria of the disease.

## Discussion

A rare congenital hamartomatous disorder called Proteus syndrome causes tissue from any of the three germinal layers to grow multifocally [1]. The term was coined by Wiedermann et al. in 1983 and further described by Cohen and Hayden [2, 3]. A somatic activating mutation in the AKT1 gene on chromosome 14q32.3 has been linked to mosaicism in Proteus syndrome. However, its detection in blood DNA samples is not consistent in all Proteus syndrome cases. Therefore, Lindhurst et al. have suggested that clinical criteria alone are sufficient to establish the diagnosis [4]. It is a sporadic disorder, although some cases exhibit familial transmission [9]. Since there isn't a specific gene test for this disorder, diagnosis mostly relies on clinical and radiological evaluation [5].

Proteus syndrome presents a spectrum of manifestations across different body systems. Soft tissue abnormalities include asymmetrical subcutaneous fat overgrowth, notably affecting the soles of the feet and palms of the hands [5]. Skeletal anomalies encompass kyphoscoliosis, abnormal vertebral bodies, segmentation defects, limb overgrowth, and macrodactyly [6]. Vascular malformations involve vascular ectasia, hemangiomata, lymphangiomata, and varicosities [10]. Additionally, visceral malformations may manifest as organomegaly, renal calculi, colonic polyps, uterine leiomyomas, pulmonary emphysema, lung scarring and consolidation, cerebral arteriovenous malformations, hydrocephalus, and schizencephaly [6]. In the case presented, the patient demonstrated soft tissue and skeletal manifestations, along with vascular malformations. Two main differentials include Klippel-Trenaunay-Weber syndrome (KTWS) and neurofibromatosis type I [6].

Participants at the NIH developed diagnostic criteria, differentials, and guidelines for evaluating parameters in 1998 [7]. Advised tests include MR abdomen and pelvis, MRI brain, and HRCT chest [6].

The diagnostic criteria for Proteus syndrome can be categorized into general and specific criteria. General criteria encompass the mosaic distribution of lesions, sporadic occurrence, and a progressive course. In contrast, specific criteria include category A, where the diagnosis is marked by the presence of cerebriform connective tissue naevus, considered pathognomonic. Category B involves asymmetric and disproportionate enlargement of either the musculoskeletal or visceral tissues, accompanied by epidermal naevus, and specific tumors such as ovarian cystadenoma and parotid monomorphic adenoma that manifest before the age of 30. Category C encompasses lung cysts, facial phenotype, vascular or lymphatic malformation, and asymmetric adipose tissue deposition, including lipomas and lymphoid hyperplasia [7].

The presence of all general criteria, along with either two category B criteria, three category C criteria, or all category A criteria, is required for the diagnosis of Proteus syndrome [5]. In our case, one category A criterion, one category B criterion, and one category C criterion were present.

The approach involves a multidisciplinary diagnosis and treatment of vascular malformations, along with thrombosis prophylaxis in high-risk situations. Due to the uneven growth of the extremities, orthopedic interventions such as braces and specialized footwear, as well as physiotherapy, are recommended. Growth regulation can be achieved through epiphysiodesis and, if necessary, surgical removal of the foot or hand rays. Radiological assessment of the chest is advised in cases of pulmonary issues and prior to surgical procedures. Lipomatous hyperplasias may require surgical resection, although recurrence is possible. Additionally, regular screening for specific tumors is recommended [8]. 

## Conclusions

The diagnosis of Proteus syndrome is significantly facilitated by imaging, which reveals the mosaic proliferation of various tissues, including adipose tissues, blood vessels, muscles, bones, and skin. It relies primarily on physical examination and imaging studies that satisfy both general and specific criteria, as there is currently no specific genetic test available. Early identification of this uncommon congenital condition can lead to an improved quality of life for the patient.
